# Commerce

**Published:** 1881-05

**Authors:** 


### COMMERCE.

What has commerce done for the world, that its history should be explored and philosophy illustrated, its claims advanced among the influences which impel civilization at so great a sacrifice of human life, and treasure ?

It has enabled man to avail himself of the peculiarities and advantages of region and climate, to establish that division of labor which tends to equalize society, to distribute the productions of the earth and to illustrate the benefit of kindly interdependence. It unites distant races of mankind, cultivates amical relations among them, encourages mutual interests, and promotes that fraternity of feeling which is the strongest bond of permanent friendship. Races, nations, and communities differing in creed, in language, in social customs and dress, when brought in communication with one another find how much there is of universal application, and improve their conditions severally, by supplying the wants of the one from the abundance of another. The friendly intercourse among nations and peoples at the farthest possible removes from one another in distance and customs, created by commerce, is surely, though slowly, it may be, revolutionizing the world. Time once was, when nations and tribes met only on the field of battle, and there was but one name for stranger and enemy. But now wherever a ship can float or sled penetrate—from the equator to the poles—the various emblems of sovereignty intermingle in harmony, and the sons of Commerce all the world over, in consulting their own interests, promote the cause of humanity and peace.

If, in seeking after the mighty influences which control human progress, the vision of the inquirer, ranging with the scope of his own ephemeral existence, censures the justice which is steadfastly pursuing its course through the countless ages, turns away bewildered by the calamities which crowd upon him and extinguish nationality in devastation and blood—let him not despair. But let him who would be discouraged and despond for humanity, and mourn for faith misplaced, for hopes betrayed, for expectations disappointed—*look back!* Have revolution and change accomplished nothing? Has there been no advance from kingly prerogative and priestly intolerance; no improvement on feudal tenure, vassalism and slavery? The end is not yet. Liberty is enlarging her bounds apace with Commerce. Let the downcast be cheered, for the Eternal Right watches over all—her king is COMMERCE, and it moves onward to overcome in its own good time.—*Sanitarian.*

